# How precise are mutation rate estimates? Comparison of different approaches to estimate de novo mutation rates

**DOI:** 10.1038/s41437-026-00852-7

**Published:** 2026-06-19

**Authors:** Xi Wang, Chaowei Zhang, Hongbo Wang, Kerry Reid, Juha Merilä

**Affiliations:** 1 Area of Ecology and Biodiversity, School of Biological Sciences, The University of Hong Kong, Hong Kong SAR, China; 2 Ecological Genetics Research Unit, Organismal and Evolutionary Biology Research Programme, University of Helsinki, Helsinki, Finland

**Keywords:** Mutation, Next-generation sequencing

## Abstract

Availability of de novo mutation rate (*µ*) estimates based on approaches that rely on bioinformatic validations has increased tremendously during the past few years, but the accuracy and precision of these estimates often remain unclear as Sanger sequencing validation of the mutations is often lacking. We used both long- and short-read sequencing data and different bioinformatic pipelines to estimate *µ*, as well as false positive (FPR) and negative (FNR) rates, for family trios of flat-headed loaches (*Oreonectes platycephalus*). By comparing estimates against PCR-verified mutations, we observed that the top-performing approach (as ranked by the F1 score of seven approaches at the same depth) still exhibited a 4% false positive rate (FPR) alongside a 12% false-negative rate (FNR). Across the remaining methods, FPR values ranged from 4–12%, and FNRs from 8–19%. Irrespective of the bioinformatic approach used, long-read data yielded consistently lower *µ* estimates than short-read data because of the larger callable genome sizes. In addition, a higher mapping depth resulted in a lower FNR. These results call for caution regarding de novo mutations without Sanger sequencing validation in non-model organisms and raise the possibility that many published *µ*-estimates, especially those based on low mapping depths, might be biased.

## Background

De novo mutations (DNMs) are spontaneous genetic alterations of profound importance in genetics and evolutionary biology (Kessler et al., 2020). Germline DNMs, when occurring, can be inherited by the next generation, adding genetic diversity that may fuel new adaptations and genetic differentiation (Goldmann et al., 2019). However, when deleterious, they can also have negative fitness consequences (Díez-del-Molino et al., 2018). Whether adaptive or deleterious, accurate estimates of DNM rates and their characteristics are important for our understanding of the evolution of mutation rates (Lynch, 2010). Moreover, accurate DNM rate estimates play an important role in answering key questions in population genetics, such as obtaining divergence time, effective population size, and migration rate estimates (Koch et al., 2019).

Recently, direct sequencing of parent-offspring (PO) trios and counting the mutations occurring over one generation has largely overtaken other methods used in DNM rate estimation (Roach et al., 2010; Wang and Obbard 2023), as selection cannot eliminate mutations within a single generation except for lethal mutations (Lynch et al., 2023). For example, Bergeron et al. (2023) quantified DNM rates across 151 parent-offspring trios in 68 species of mammals, fishes, birds and reptiles. However, given that the expected number of DNMs per generation is low and that sequencing and mapping errors complicate identification, robust identification of true DNMs remains challenging (Yoder and Tiley, 2021). Long-lived species tend to have more DNMs per generation (Wang and Obbard, 2023) and, therefore, overestimation of their number of DNMs may not have as much impact on their DNM rate compared to short-lived species. With far fewer per-generation DNMs, short-lived taxa experience disproportionate DNM rate estimation bias when the false positives (FPs) are counted as true DNMs - even a single FP can markedly skew the results. Thus, minimizing FPs is essential for the accurate detection of DNMs via the PO-trio method, particularly in short-lived species (Bergeron et al., 2022). Many recent studies have relied on bioinformatic strategies seeking to minimize FPs, rather than using Sanger sequencing for validation. Bergeron et al. (2023) proposed to manually curate DNMs using visualization software (e.g., IGV; Robinson et al., 2011), which has now been widely applied in recent studies (e.g., Zhang et al., 2023; Versoza et al., 2025; Wooldridge et al., 2025; Zhang et al., 2025). In fact, a compilation of data from published studies (*n* = 38) that have used the PO-trio method to estimate the DNM rate for Animalia, excluding human disease research (Table S1, Supplemental Material), reveals that only 39% of these verified DNMs were by experimental approaches (e.g., Sanger sequencing), the rest relying on bioinformatic validation only. However, the accuracy of software-based bioinformatic validation has not yet been evaluated, leaving open the question of whether this approach is reliable without further Sanger sequencing validation.

The majority of DNM research based on PO-trios has applied short-read sequencing (SRS; e.g., Illumina HiSeq, DNBseq, BGISEQ500) as the sequencing strategy because of its cost-effectiveness and low per-base error rate (e.g., Malinsky et al., 2018; Bergeron et al., 2023; Burda and Konczal, 2023; Liang et al., 2024). However, SRS methods face challenges in accurately capturing highly complex genomic regions, such as repetitive sequences and regions with indels or structural variants (Ashley, 2016). Compared to SRS, long-read sequencing (LRS) can generate >10 kb reads, which has been proven to improve the detection of indels and other structural variants (Chaisson et al., 2015; Logsdon et al., 2020; Pauper et al., 2021). Methodological differences may directly lead to divergent performance in DNM detection and in the calculation of callable genome size, thereby affecting the DNM rate estimate. Specifically, LRS may expand the callable genome size to include previously intractable complex genomic regions, thereby increasing the total number of detectable DNMs. Nevertheless, DNM rate estimation relies on de novo point mutations (i.e., single-nucleotide variants, SNVs), which have rarely been evaluated in LRS research (but see Noyes et al., 2022; Kucuk et al. 2023; Porubsky et al., 2025). Beyond sequencing strategy, sequencing depth is equally critical for the robust identification of DNMs with minimal false positives and false negatives (Tatsumoto et al., 2017). Determining sequencing depth is a trade-off: higher depth improves the accuracy of variant genotyping, but demands greater costs, and the efficacy of this investment remains uncertain. Hence, addressing this knowledge gap is urgently needed.

The main aims of this study were twofold. First, to compare the performance of DNM validation - with and without Sanger sequencing in controlling FPs. Second, to compare the performance of various sequencing strategies and sequencing depths on calling DNMs and to estimate the false positive and negative rates of these approaches. To this end, we used trio data from a diploid freshwater fish, the flat-headed loach (*Oreonectes platycephalus*), owing to access to a high-quality, well-defined, chromosome-anchored, haplotype-resolved reference genome (Wang et al., 2025).

## Methods

### Sample collection and DNA extraction

The parental loaches for this study were collected from Lung Fu Shan (22.2785°N, 114.1330°E), Hong Kong, in December 2022 and transported to the aquarium facilities of the University of Hong Kong. The offspring were generated by placing one female and one male individual in 75 L (50*30*50 cm) aquaria allowing them to reproduce naturally. A total of six offspring were produced, and once the larvae were ca. 100 days old, both the parents and the offspring were euthanized by overanesthetizing with MS-222 and preserved in 95% ethanol for DNA extraction. Genomic DNA was extracted from the pectoral fins using the *DNeasy Blood and Tissue Kit* (Qiagen, Germany). The DNA quality of the samples was evaluated with *NanoDrop*^*TM*^ *One spectrophotometer* (Thermo Fisher, MA, USA) and *Qubit dsDNA HS Assay Kit* with *Qubit*^*TM*^ *4.0* (Invitrogen, CA, USA). Once the DNA samples were sent to the sequencing company, they conducted an additional sample quality evaluation to ensure that the samples met the requirements of library construction.

### Sequencing and mapping

The eight loach DNA samples were sequenced at Annoroad Gene Technology Co., Ltd (Beijing, China) using PacBio Revio^TM^ HiFi (High Fidelity) technology for long-read sequencing (LRS). Briefly, 5 µg of DNA from each sample was utilized for *SMRT (Single-Molecule Real-Time)* library preparation with the *SMRTbell Express Template Prep Kit 2.0* (Pacific Biosciences Inc., CA, USA). The DNA molecules in the *SMRTbell* library were sequenced in real time by DNA polymerase, targeting 30x depth per sample. The Revio platform includes a built-in DeepConsensus v1.2.0 for correcting errors in Circular Consensus Sequencing (CCS) reads. The HiFi reads were aligned to the loach *HapA* reference genome, which was also sampled from Lung Fu Shan (Wang et al., 2025) using Minimap2 v2.28 (Li, 2018) with the parameters “-ax map-hifi -a -k 19 -O 5,56 -E 4,1 -B 5 -z 400,50 -r 2k --eqx --secondary=no” to improve mapping quality following Zhou et al. (2022). For short-read sequencing (SRS), the eight samples were sent to Berry Genomics Co., Ltd (Beijing, China) using the DNBSEQ^TM^ T7 platform (MGI Tech Co., Ltd., China). For each of the eight samples, 1 µg of DNA was used to prepare a PCR-free library. The DNBSEQ sequences were mapped to the loach *HapA* reference genome (GenBank: GCA_045838175.1; Wang et al., 2025) using BWA v0.7.17 (Li, 2013) with default settings, following quality control with fastp v0.23 (Chen et al., 2018). Before variant calling, duplicate reads in the SRS BAMs were marked and filtered using SAMtools v1.21 (Li et al., 2009). The average mapping depth was ~30x for LRS and ~45x for SRS. To avoid mapping depth affecting comparison, the SRS sequences were downsampled to ~30x (Table S2).

### Variant calling and candidate DNM identification

The difference between LRS and SRS for variant calling lies in the input BAM files: LRS uses sorted BAM files, whereas SRS uses BAM files with duplicates marked. Variant calling was performed via three approaches - GATK v4.5.0.0 (McKenna et al., 2010), DeepVariant v1.6.1 (Poplin et al., 2018) and DeepTrio v1.6.1 (Kolesnikov et al., 2021). GATK HaplotypeCaller was applied to call variants for each individual from the mapped BAM files for both LRS and SRS. The obtained genotypes were combined through GATK CombineGVCFs. After variant calling, SNVs were genotyped by GATK GenotypeGVCFs and picked out by GATK SelectVariants, followed by hard filtering by GATK VariantFiltration with parameters “MQRankSum < −12.5||SOR > 3.0||FS > 60.0 || ReadPosRankSum < −8.0||MQ < 40.0||QD < 2.0” as advised in Bergeron et al. (2022). For the DeepVariant pipeline, we applied it to call variants of each individual and merged the variants via GLnexus v1.4.1 (Yun et al., 2021) with default settings. The DeepTrio is a deep learning-based trio variant caller built on top of DeepVariant, which calls variants of each pedigree trio and the obtained gVCFs were merged by GLnexus utilizing the parameter of “--config DeepVariant_unfiltered”. In addition to calling variants from LRS and SRS, we applied a hybrid DeepVariant model by combining LRS and SRS as a new dataset using SAMtools’ merge function. To test the impact of mapping depth, we also downsampled the hybrid dataset to different mapping depths - 15x, 30x (used for the method comparison), 45x, 60x and 75x, respectively. The variants of the five hybrid datasets were called using DeepVariant with the parameter “--model_type HYBRID_PACBIO_ILLUMINA”.

The variants in each parent-offspring trio VCF file were filtered to a subset of single-nucleotide variants (SNVs) to identify de novo mutation (DNM) candidates by VCFtools v0.1.17 (Danecek et al., 2011). To obtain reliable DNM candidates, a series of filters was applied to the SNVs following the Sticklebacks pipelines (Zhang et al., 2023):

1. Mendelian violation - keep sites where both parents are homozygous for the reference allele (0/0) while the offspring is heterozygous (0/1) as mutation sites, excluding the sex-determining region (i.e., 0–14 Mb at Chr01) of the loach genome (Wang et al., 2025).
2. To correct genotype errors, we applied filtering based on genotype quality (GQ) generated by the variant calling software. GQ thresholds were set differently for each approach: for GATK, we used a threshold of 50 for parents and 80 for offspring, whereas for other methods we used 30 for parents and 10 for offspring, according to the GQ distribution shown in Figure S1.
3. Sites with low read depth (DP) may reflect sequencing or genotype errors while high DP would happen in a misalignment. Therefore, the candidate DNMs were called with DP between 0.5*DP_mean_ to 2*DP_mean_ (Table S2).
4. To ensure the real homozygosity for the two parents, the allelic depth (AD) filter was applied (AD_1_ > 0 and AD_0_ ≤ 1).
5. For germline mutations, the allelic balance (AB) is expected to have a peak around 50% while the peak around 20% is likely to be indicative of somatic mutations (Besenbacher et al., 2015). As a result, the sites with an allelic balance between 0.3 and 0.7 were considered as candidate DNMs.
6. Considering DNMs are expected to occur with low probability, the clustered mutation sites where more than two DNM candidates were observed within 100 bp were double checked via IGV v2.18.4 (Robinson et al., 2011) to confirm whether these candidates were caused by error-prone DNA polymerases. If not, they would be regarded as false positives caused by misalignment.
7. DNM candidates were further confirmed using IGV, where the BAM files of every parent-offspring trio were checked to ensure the raw mapped reads supported the genotype. Only when all parental reads were homozygous and the offspring satisfied all the above-described criteria, the candidate DNM was accepted (Figure S2, Supplemental Material).

### DNM validation and annotation

All candidate DNMs were attempted to be validated by Sanger sequencing of the family trio. Specific primers were designed using Primer-BLAST (Ye et al., 2012) and synthesized by Tsingke Biotechnology Co., Ltd. (Beijing, China). PCRs for the validation (see Supplemental Material) were performed using Veriti™ Thermal Cycler (Applied Biosystems, MA, USA). Paired-end Sanger sequencing was performed by Sangon Biotech Co., Ltd. (Shanghai, China), and the traces were analyzed using Geneious v2025.0.3 (Kearse et al., 2012). The true DNM would be with all parental reads that were homozygous, while the offspring reads were heterozygous (Supplemental Material).

The candidate DNMs were annotated to genomic locations (i.e., within exons, introns, or intergenic regions) based on the previously published loach genome assembly and annotation (Wang et al., 2025). In addition, mutation spectra were analyzed according to the alternative and reference alleles. The validated DNMs were then divided into transversions (Tv: A > C, A > T, C > A, and C > G) and transitions (Ts: A > G and C > T with a special inspection on the C > T mutations on the CpG sites).

### De novo mutation rate estimation

The germline de novo mutation per-site per-generation for each family trio (*µ*_*trio*_) can be calculated as:

1 $${\mu }_{{trio}}\,=\,\frac{N}{2\,\times \,{CG}\,\times \,(1\,-\,{fFNR})}$$

where *N* was the number of true DNM of each trio; CG was the callable genome; fFNR is the filtering false-negative rate.

The callable genome (CG) was the portion of the genome that could detect candidate DNMs. This can be calculated by selecting every position in the gVCF files of each trio for which both parents were homozygous for the reference allele (0/0), as well as all three individuals passed DP filters (i.e., 0.5*DP_mean_ to 2*DP_mean_; Bergeron et al., 2021) and passed hard filtering for the GATK approach. The filtering false-negative rate was the proportion of true DNMs that could have been filtered out by allelic balance filters, which can be calculated as:

2 $${fFNR}\,=\,\frac{{n}_{{true}\,{heterozygotes}\,{removed}\,{by}\,{AB}}}{{n}_{{true}\,{heterozygotes}}}$$

where n_true heterozygotes_ was the total number of heterozygous sites that the offspring was 0/1 with one parent carrying 0/0 and the other having 1/1; and n_true heterozygotes removed by AB_ was those true heterozygotes that did not pass the above AB filter (AB < 0.3 and AB > 0.7)

The germline de novo mutation per-site per-generation for the pedigree was then estimated as:

3 $$\mu \,=\,\frac{{\sum }_{i=1}^{n}{N}_{i}}{{\sum }_{i=1}^{n}[2\,\times \,{{CG}}_{i}\,\times \,(1\,-\,{fFN}{R}_{i})]}$$

where *i denotes* the given offspring.

For each approach and mapping depth, the 95% confidence intervals of *µ* were obtained using the bootstrap method with 10,000 resampling replicates.

### Comparison of different approaches

The candidate and true DNMs were marked in relation to the approach and the intersection was summarized through an UpSet plot (Lex et al., 2014) via UpSetR (https://gehlenborglab.shinyapps.io/upsetr/). The false positive rate (FPR) and false-negative rate (FNR) of each approach were calculated, where FPR was the percentage of candidate DNMs that could not be validated by Sanger sequencing and FNR was the percentage of true DNMs that were missed by the approach but counted in the union of all approaches. Based on the FPRs and FNRs, we calculated the F1 score for each approach, an evaluation metric that quantifies an approach’s accuracy (Powers, 2011). F1 score combines the precision and recall scores of an approach - precision is affected by false positives while recall is affected by false negatives:

4 $${\rm{Precision}}=\frac{N}{N\,+{n}_{{false}\,{positive}}}$$

5 $${\rm{Recall}}=\frac{N}{N\,+{n}_{{false}\,{negative}}}$$

6 $${\rm{F}}1=\frac{2\,\times \,{Precision}\,\times \,{Recall}}{{Precision}\,+\,{Recall}}$$

Paired-samples t-tests were conducted for *µ* across the approaches. In addition, a linear model was built to assess the impact of the mapping depth on FNR with the downsampled hybrid dataset.

## Results

### Variants overview

**Fig. 1:**
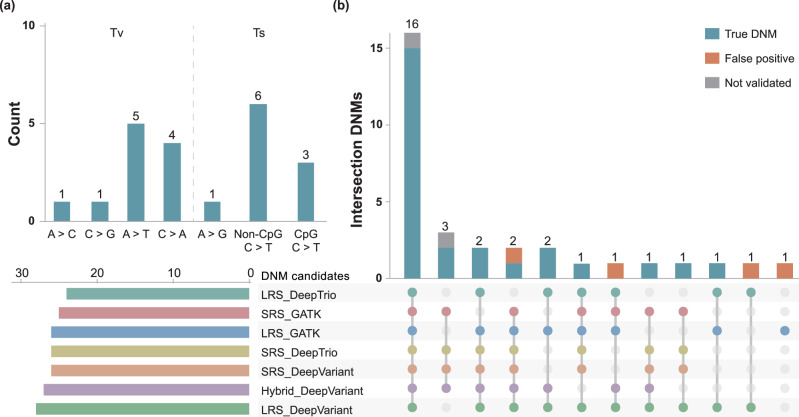
Mutation spectra and the intersection of different approaches of the loach DNM candidates. **a** Mutation spectra of unique DNMs validated by Sanger sequencing. **b** UpSet plot depicting the intersection of different approaches in the identification and validation of DNMs with the proportion of true DNMs, false positives and not validated (DNMs failed for primer design) shown below each category.

We utilized an average mapping depth of ~30x for LRS, SRS and hybrid dataset from the loach (genome size: 565.68 Mb) pedigree consisting of two parents and six offspring (Table S2 and Figure S2) to call variants with seven approaches - LRS_DeepVariant, LRS_DeepTrio, LRS_GATK, SRS_DeepVariant, SRS_DeepTrio, SRS_GATK and Hybrid_DeepVariant. After a series of filtering and manual IGV checking (Supplemental Material), only 32 candidate DNMs (Table S3) were detected. We then designed primers and validated these candidate DNMs through Sanger sequencing (Supplemental Material). Considering candidate DNMs confirmed by Sanger sequencing as true mutations, a total of 26 true DNMs (Table S3) were identified in the offspring (2–6 DNMs per individual, mean DNM = 4.3) with 1, 11, and 14 DNMs respectively found in the exonic, intronic, and intergenic regions, among which 3 were within repeat regions, including 3 shared DNMs among siblings (Table S3). Among 21 unique validated DNMs (shared DNMs only counted once), 47.61% were transitions (Ts) and 52.38% were transversions (Tv), resulting in a Ts:Tv ratio of 0.91 (Table S3). The most common mutation type was C:G to T:A transition, with one-third located on CpG sites (Fig. 1a). Additionally, four false positives were identified by Sanger sequencing (Supplemental Material) - all on introns, with one on repeat regions. Two candidates were not validated due to failed PCR amplification; both were located outside repeat regions.

Of the seven different approaches used (Table 1), the DeepVariant-based methods outperformed others with the highest accuracy, reflected in low false positive and negative rates (Table 1). Approaches such as SRS_Deeptrio also demonstrated balanced performance but LRS_Deeptrio had a higher false-negative rate than the DeepVariant-based methods (Table 1). In contrast, GATK-based methods, regardless of using LRS or SRS, faced significant sensitivity challenges (FNR > 15%) and struggled with specificity (FPR ~ 10%; Table 1). False-negative sites were due to allelic balance ratios falling outside the 0.3–0.7 threshold. The SRS-based methods exhibited significantly smaller CG sizes than those of the LRS-based methods (Table 1 and Figure S3, Supplemental Material). When the intersection of DNM candidates across approaches was computed (Fig. 1b), it yielded 16 DNMs with 0% FPR. On the contrary, if a DNM candidate was called by only one or two approaches out of the total seven, it was a false positive (Fig. 1b), suggesting that the use of a broad combination of approaches results in more accurate and reliable DNM detection.

**Table 1:** Comparison of the performance of different approaches to call de novo mutations from flat-headed loach family trios.

	LRS_DeepVariant	LRS_Deeptrio	LRS_GATK	SRS_DeepVariant	SRS_Deeptrio	SRS_GATK	Hybrid_30x (DeepVariant)
Candidate DNMs	28	24	26	26	26	25	27
True DNMs^a^	24	21	22	23	23	21	23
False positive sites^b^	3	2	3	1	1	2	2
Unknown^c^	1	1	1	2	2	2	2
False positive rate^d^	0.11( ± 0.07)	0.09( ± 0.06)	0.12( ± 0.07)	0.04( ± 0.04)	0.04( ± 0.04)	0.09( ± 0.06)	0.08( ± 0.06)
False-negative rate^e^	0.08( ± 0.07)	0.19( ± 0.09)	0.15( ± 0.08)	0.12( ± 0.07)	0.12( ± 0.07)	0.19( ± 0.09)	0.12( ± 0.07)
Mean Callable genome	453945298	451051569	458468975	439533971	439579519	437892547	455907144

All approaches are based on data at 30x depth.

^a^True DNMs: DNMs validated by Sanger sequencing;

^b^False positive sites: variants were validated as homozygous in offspring;

^c^Unknown: failed PCR amplification;

^d^False positive rate (FPR) = False positive sites/(True DNMs + False positive sites);

^e^False-negative rate (FNR) = 1- True DNMs called by a certain approach/ union of True DNMs called by all approaches (i.e., 26). Values in parentheses are standard deviation estimates for FPR and FNR.

### Effect of mapping depth on DNM detection

**Fig. 2:**
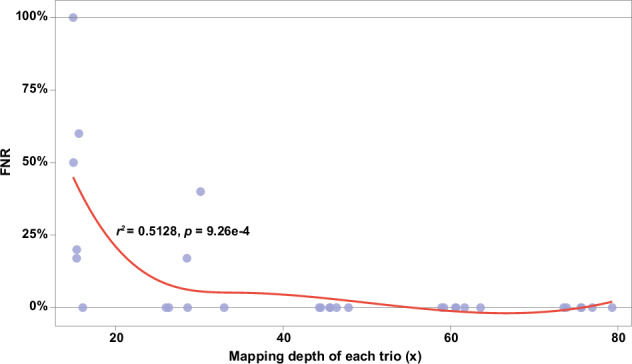
The impact of the mapping depth of the Hybrid datasets using DeepVariant for each trio on the false-negative rate (FNR). Each data point corresponds to each trio at different mapping depths. The red curve represents the predicted trend line from a one-knot spline regression model (knot position: *x* = 35) fitted to FNR values across different mapping depths.

Mapping depth also affected the performance of calling DNMs. We downsampled the hybrid (SRS + LRS) sequencing data to mapping depths of 15x, 30x, 45x, 60x, and 75x, and applied DeepVariant to call variants in the hybrid datasets. There was a clear inverse relationship between mapping depth and FNR (*r*^*2*^ = 0.51, *F*_*25*_ = 6.578, *p* < 0.05; Fig. 2 and Table S4). At lower depths, the FNR was highly variable, even once reaching 100%. However, as the depth increased beyond ~45x, the FNR decreased and stabilized to near 0% (Fig. 2). For FPR, 15x and 30x datasets detected one more site than the higher depths.

### Comparison of different approaches

**Fig. 3:**
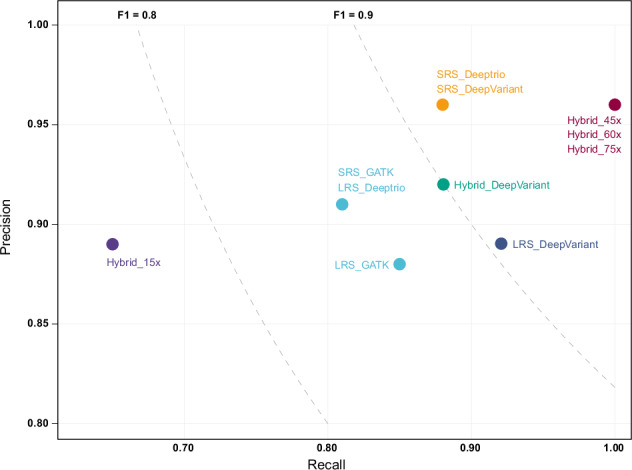
Precision-recall distribution of variant calling methods, with F1 scores (harmonic mean of precision and recall) represented by iso-contour lines. Ideal performance corresponds to the upper-right quadrant, where both precision (x-axis) and recall (y-axis) are maximized. Methods closer to the upper-right corner achieve superior balance between accuracy and sensitivity. Points for the Hybrid_DeepVariant method under a gradient of mapping depth were included. Points sharing the same color indicate identical F1 scores.

For a quantitative comparison of the method performance, we calculated the F1 score, a quantity composed of “recall” measuring the ability of a method to detect true positive mutations, and “precision” measuring the proportion of detected mutations that are true positives (Powers, 2011). Overall, when mapping depth reached 45x or above, the hybrid methods achieved the highest F1 score (0.98) (Fig. 3 and Table S5). Down to ~30x, the F1 score of the hybrid method remained over 0.9; down to ~15x, it dropped to 0.76 (Fig. 3 and Table S5). As for SRS, the DeepTrio and DeepVariant approaches reached the same performance (F1 score = 0.92) but the F1 score of the GATK approach was below 0.9 (Fig. 3 and Table S5). For the LRS data, the F1 score of the DeepVariant approach was higher (0.91) than that of the other two methods (0.86; Fig. 3 and Table S5). Use of DeepVariant for variant calling appeared to be superior to other methods, regardless of the sequencing method (Fig. 3).

### Mutation rate estimates

**Fig. 4:**
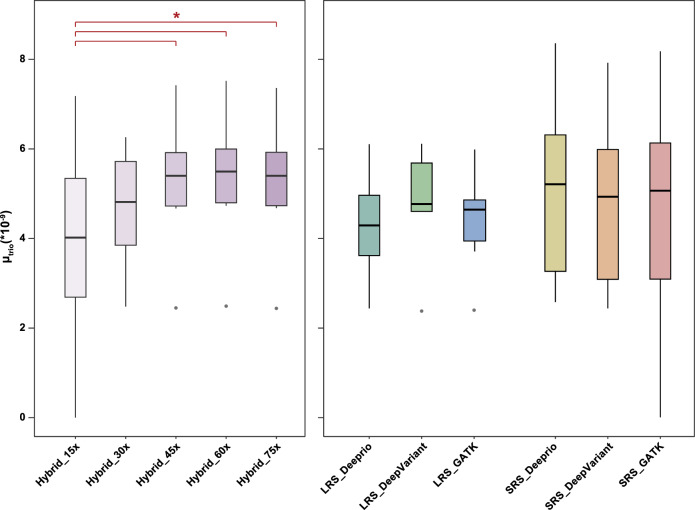
Mutation rate estimates (*µ*) obtained under different mapping depths or variant calling approaches. Estimates obtained under different mapping depths via the Hybrid_DeepVariant method (left) and using different variant calling approaches at 30x (right). Boxes represent interquartile ranges (IQR), horizontal lines denote medians, whiskers extend to 1.5×IQR, and dots indicate outliers. Asterisks (*) indicate statistically significant differences based on *t*-tests.

As the depth increased to ~45x or above, the average *µ* was over 5.20*10^−9^ /bp/generation, while the rest of the mean estimates were less than 5.00*10^−9^, no matter what variant calling software was used (Fig. 4 and Table S6). The *µ* estimated via the Hybrid_15x approach was significantly lower than that obtained with the Hybrid_45x (paired *t*-test, *t*_*5*_ = 2.20, *p* = 0.04), Hybrid_60x (*t*_*5*_ = 2.33, *p* = 0.03) and Hybrid_75x (*t*_*5*_ = 2.17, *p* = 0.04) approaches, reflecting the impact of its high FNR (Table 1 and Fig. 2). The germline de novo mutation rate estimates for LRS data yielded consistently lower estimates than SRS data, but not significant (Welch *t-test*, *t*_*2*_ = 3.93, *p* = 0.09; Fig. 4 and Table S6). However, significant differences were found in CG (ANOVA, *F*_*4*_ = 7.41, *p* = 0.001; Figure S3 and Table S6, Supplemental Material). Applying the *µ* estimated by the mean of Hybrid 45x, 60x and 75x (with identical F1 scores), the final best estimate of de novo mutation rate of the flat-headed loach was 5.23*10^−9^/bp/generation (Table S6).

## Discussion

We compared the effectiveness of validation approaches with and without Sanger sequencing and found that the sole use of bioinformatics methods was insufficient to filter out false positives, leading to a substantial overestimation of mutation rates. Given that more than half of the earlier studies (*n* = 23 out of 38) estimating mutation rates we reviewed (Table S1, Supplemental Material) have relied solely on bioinformatics-based validation, it is likely that the mutation rates in these studies have been overestimated. Given that bioinformatics visualization tools seem to lack precision, it is essential to estimate the FPR through Sanger sequencing. However, designing Sanger sequencing experiments in non-model species can be challenging, especially at repeat regions (Bergeron et al., 2022; Versoza et al., 2025). Yet, even if it is difficult and impractical to examine each candidate DNM in multiple pedigree studies with numerous individuals, a subset of candidate DNMs could be examined to estimate FPR.

Long-read sequencing (LRS) has been widely recognized as an improved strategy for traversing repetitive regions, thereby enhancing variant detection, particularly for structural variant calling (Mahmoud et al., 2019). However, DNM rates for SNVs have seldom been estimated from LRS data (but see Noyes et al., 2022; Kucuk et al., 2023; Porubsky et al., 2025; López-Cortegano et al., 2025). Here, we evaluated whether LRS would outperform short-read sequencing (SRS) in DNM identification. An average of 22.33 true point DNMs were identified from LRS data using three variant calling softwares, which was equal to true DNMs identified by SRS and confirmed with Sanger sequencing, suggesting that using LRS data to call de novo point mutations is not necessarily better than using SRS data. This result is in contrast to those of earlier studies, which have estimated higher numbers for LRS than for SRS data (Noyes et al., 2022; Porubsky et al., 2025). However, all these earlier studies were conducted on humans with advanced reference genomes (Nurk et al., 2022), well-characterized variant databases (Karczewski et al., 2020), and advanced sequencing technology utilizing multiple-generation trio data (Porubsky et al., 2025). In contrast, non-model organisms often lack gapless telomere-to-telomere (T2T) reference genomes and benchmarks, making it more challenging to fine-tune filters effectively. Consequently, issues related to FNR and FPR in DNM rate estimation are likely to be more pronounced in studies of non-model species. Therefore, a tailored approach is essential when conducting DNM analyses in non-model species. For trio-based studies, calling variants against a high-quality and closely related de novo genome assembly may outperform a reference genome constructed from a population that is highly diverged from the focal samples, as the closely related assembly contains fewer misassemblies and therefore yields a larger callable genome size. With the rapid development of sequencing and genome assembly technology, researchers now have greater opportunities to improve data accuracy, enhance variant detection methods, and develop species-specific pipelines that can better account for the unique genomic features of non-model organisms. Hence, limited reference data can lead to higher FNR and FPR, thereby affecting the accuracy of DNM detection and rate calculations.

We note that GATK (McKenna et al., 2010) was designed for SRS data, which may have influenced its performance on LRS data. However, the GATK dataset showed an equal F1 score (Fig. 3 and Table S5) between LRS and SRS data in this study. In addition, deep-learning-based variant calling tools, such as DeepVariant v1.6.1 (Poplin et al., 2018) and DeepTrio v1.6.1 (Kolesnikov et al., 2021), are known to yield higher F1 scores than traditional algorithm-based tools (Abdelwahab and Torkamaneh, 2025). In our study, GATK consistently yielded the lowest F1 scores, aligning with the view emerging from the review by Abdelwahab and Torkamaneh (2025). Since no other study has utilized DeepVariant for SRS DNM calling in non-model species, further investigation into the performance of different softwares is warranted. Furthermore, although DeepTrio was specifically designed for variant calling in parent-offspring trios, it achieved lower F1 scores than DeepVariant in the LRS dataset. This may be due to the models being trained predominantly on human data, which could limit their effectiveness when applied to non-model species. Regardless, our results suggest that, at the same mapping depth (30x), DeepVariant may be the best choice for DNM calling, outperforming the other software tested. Besides the sequencing strategy and variant calling approaches, mapping depth was also found to be an important factor affecting DNM detection. By combining LRS and SRS data, we obtained a hybrid sequencing dataset for DNM calling and downsampled it to a gradient of depths. While 15x depth has been historically recommended for reliable SNV calling (Fumagalli et al., 2013), our analysis revealed that this depth is insufficient for robust DNM detection, as evidenced by a high FNR (0.35) and the lowest F1 score (0.76) across all tested depths (Fig. 3 and Table S5). This discrepancy likely stems from the stricter statistical thresholds required to distinguish rare, true DNMs from background sequencing noise, which demand greater read coverage than those required for common SNVs. Most of the PO-trio-based DNM studies sequenced at 30x to 50x (e.g. Bergeron et al., 2023; Zhang et al., 2023; Zhang et al., 2025), while we found the performance of 45x (F1 score = 0.98) was much better than 30x (F1 score = 0.86; Fig. 3 and Table S5). Notably, further increases in depth to 60x or 75x did not yield additional gains in accuracy, with performance metrics remaining identical to those at 45x (Fig. 3 and Table S5). This plateau effect indicates that 45x represents a cost-effective benchmark for high-confidence DNM detection in hybrid sequencing workflows, providing an optimal balance among sensitivity, specificity, and resource expenditure, substantially higher than the previously recommended depth (35x) by Bergeron et al. (2022) for the SRS method.

The mutation rate estimate (*µ*) depends not only on the number of DNMs but also on the callable genome size (CG) and the filtering false-negative rate (fFNR). Notably, SRS-based approaches (SRS_Deeptrio, SRS_DeepVariant, SRS_GATK) yielded consistently higher *µ* than the LRS-based approaches (LRS_Deeptrio, LRS_DeepVariant, LRS_GATK; Fig. 4 and Table S6). To dissect the factors underlying this discrepancy, one-way ANOVA was performed on CG and fFNR obtained under different mapping depths or using different approaches at 30x (Figure S3 and Table S6, Supplemental Material). The LRS-based approaches consistently achieved the highest callable genome sizes, with no significant differences among them (Figure S3), whereas SRS-based methods exhibited significantly smaller CG sizes. This observation aligns with the known strengths of long-read sequencing in spanning repetitive genomic regions and resolving complex structural variants, thereby reducing gaps in the callable genome (Chaisson et al., 2015). While the number of DNMs detected by SRS- and LRS-based approaches was nearly equal in this study, and the fFNR values were similar (except for SRS_Deeptrio), the differences in their *µ* estimates may stem from variations in CG size. However, the determinants of *μ* exhibited distinct patterns across mapping depths: CG size appeared insensitive to mapping depth, while fFNR was strongly depth-dependent (Figure S3 and Table S6). The 30x hybrid sequencing dataset (Hybrid_30x) achieved the largest callable genome size, significantly outperforming hybrid datasets generated at other sequencing depths (Figure S3 and Table S6). Analysis of fFNR metrics further revealed significant differences across different mapping depths: the 15x hybrid dataset (Hybrid_15x) exhibited the highest fFNR (Figure S3 and Table S6), reflecting the challenges of variant detection at low depth. In contrast, hybrid datasets with higher mapping depths (Hybrid_45x, Hybrid_60x, Hybrid_75x) had the lowest fFNR values (Figure S3 and Table S6), demonstrating that increased sequencing depth directly enhances the sensitivity of variant calling. These results demonstrate that the choice of sequencing approach and depth profoundly impacts the *µ* estimate. LRS-based approaches excel at maximizing callable genome size, whereas sequencing at higher depths minimizes false negatives, providing a framework for selecting sequencing strategies tailored to specific experimental goals, whether prioritizing genome coverage, sensitivity, or cost efficiency.

Although DNM rates are now available for many non-model species (Wang and Obbard 2023), there are only 14 estimates for teleost species, many of which are based on a fairly small number of mutations (Table S7). We identified a total of 26 DNMs in the loach pedigree with the de novo germline mutation rate of 5.23 × 10^−9^ /bp/generation, which was at an intermediate level compared to other germline mutation rate estimates for fish (Figure S4 and Table S7). However, given that few of these other studies have validated DNMs with Sanger sequencing (but see Feng et al., 2017; Burda and Konczal, 2023; Sendell-Price et al., 2023) and different variant calling pipelines have been used, it is hard to say what the true positioning of our estimate is relative to the published estimates.

Finally, we note that the unusual transition/transversion ratio observed in our study is unlikely to be attributable to the small number of parent-offspring trios (*n* = 6) and mutations screened (*n* = 26), or to problems with SNP calling. This is because we screened another family with 13 parent-offspring trios and 44 mutations using SRS data and observed a very similar transition/transversion ratio (0.913; Supplemental Material). Hence, it seems likely that this low ratio is a species-specific peculiarity. In fact, similar low ratios (0.95-0.98) have also been observed in two species of Pacific salmon (Smith et al., 2005). Nevertheless, given the limited number of mutations screened in this study, further studies comparing different methodological approaches for DNM detection using a larger number of parent-offspring trios are warranted.

## Conclusions

Based on our findings, accurate estimation of DNM rates for non-model species remains a complex challenge, influenced by multiple factors. Bioinformatics-based validation alone tends to overestimate mutation rates due to false positives, emphasizing the importance of extra validation such as Sanger sequencing. Sequencing depth plays an important role in reducing false-negative and false positive rates. Further validation of published DNM rate estimates for non-model species is required before these estimates will be truly reliable for population genetic studies.

## Supplementary information

Supplemental Tables

Supporting information

## Data Availability

Sequencing data has been stored in the National Center for Biotechnology Information (NCBI) SRA database (SRR30361786~SRR30361787, SRR34216341~SRR34216354) under the BioProject PRJNA1128407. Scripts were available on GitHub https://github.com/heiwong2/DNMrate_loach.git.

## References

[CR1] Abdelwahab O, Torkamaneh D (2025) Artificial intelligence in variant calling: a review. Front Bioinform 5: 1574359. 40337525 10.3389/fbinf.2025.1574359 PMC12055765

[CR2] Ashley EA (2016) Towards precision medicine. Nat Rev Genet 17(9):507–522. 27528417 10.1038/nrg.2016.86

[CR3] Bergeron LA, Besenbacher S, Bakker J, Zheng J, Li P, Pacheco G et al. (2021) The germline mutational process in rhesus macaque and its implications for phylogenetic dating. GigaScience 10(5):giab029. 33954793 10.1093/gigascience/giab029 PMC8099771

[CR4] Bergeron LA, Besenbacher S, Turner T, Versoza CJ, Wang RJ, Price AL et al. (2022) The Mutationathon highlights the importance of reaching standardization in estimates of pedigree-based germline mutation rates. eLife 11: e73577. 35018888 10.7554/eLife.73577 PMC8830884

[CR5] Bergeron LA, Besenbacher S, Zheng J, Li P, Bertelsen MF, Quintard B et al. (2023) Evolution of the germline mutation rate across vertebrates. Nature 615(7951):285–291. 36859541 10.1038/s41586-023-05752-y PMC9995274

[CR6] Besenbacher S, Liu S, Izarzugaza JMG, Grove J, Belling K, Bork-Jensen J et al. (2015) Novel variation and de novo mutation rates in population-wide de novo assembled Danish trios. Nat Commun 6: 5969. 25597990 10.1038/ncomms6969 PMC4309431

[CR7] Burda K, Konczal M (2023) Validation of machine learning approach for direct mutation rate estimation. Mol Ecol Resour 23(8):1757–1771. 37486035 10.1111/1755-0998.13841

[CR8] Chaisson MJ, Huddleston J, Dennis MY, Sudmant PH, Malig M, Hormozdiari F et al. (2015) Resolving the complexity of the human genome using single-molecule sequencing. Nature 517(7536):608–611. 25383537 10.1038/nature13907 PMC4317254

[CR9] Chen S, Zhou Y, Chen Y, Gu J (2018) fastp: an ultra-fast all-in-one FASTQ preprocessor. Bioinformatics 34(17):i884–i890. 30423086 10.1093/bioinformatics/bty560 PMC6129281

[CR10] Danecek P, Auton A, Abecasis G, Albers CA, Banks E, DePristo MA et al. (2011) The variant call format and VCFtools. Bioinformatics 27(15):2156–2158. 21653522 10.1093/bioinformatics/btr330 PMC3137218

[CR11] Díez-del-Molino D, Sánchez-Barreiro F, Barnes I, Gilbert MTP, Dalén L (2018) Quantifying temporal genomic erosion in endangered species. Trends Ecol Evol 33(3):176–185. 29289355 10.1016/j.tree.2017.12.002

[CR12] Feng C, Pettersson M, Lamichhaney S, Rubin C-J, Rafati N, Casini M et al. (2017) Moderate nucleotide diversity in the Atlantic herring is associated with a low mutation rate. eLife 6: e23907. 28665273 10.7554/eLife.23907 PMC5524536

[CR13] Fumagalli M, Vieira FG, Korneliussen TS, Linderoth T, Huerta-Sánchez E, Albrechtsen A et al. (2013) Quantifying population genetic differentiation from next-generation sequencing data. Genetics 195(3):979–92. 23979584 10.1534/genetics.113.154740 PMC3813878

[CR14] Goldmann JM, Veltman JA, Gilissen C (2019) De novo mutations reflect development and aging of the human germline. Trends Genet 35(11):828–839. 31610893 10.1016/j.tig.2019.08.005

[CR15] Karczewski KJ, Francioli LC, Tiao G, Cummings BB, Alföldi J, Wang Q et al. (2020) The mutational constraint spectrum quantified from variation in 141,456 humans. Nature 581(7809):434–443. 32461654 10.1038/s41586-020-2308-7 PMC7334197

[CR16] Kearse M, Moir R, Wilson A, Stones-Havas S, Cheung M, Sturrock S et al. (2012) Geneious basic: an integrated and extendable desktop software platform for the organization and analysis of sequence data. Bioinformatics 28(12):1647–1649. 22543367 10.1093/bioinformatics/bts199 PMC3371832

[CR17] Kessler MD, Loesch DP, Perry JA, Heard-Costa NL, Taliun D, Cade BE et al. (2020) De novo mutations across 1,465 diverse genomes reveal mutational insights and reductions in the Amish founder population. Proc Natl Acad Sci 117(5):2560–2569. 31964835 10.1073/pnas.1902766117 PMC7007577

[CR18] Koch EM, Schweizer RM, Schweizer TM, Stahler DR, Smith DW, Wayne RK et al. (2019) De novo mutation rate estimation in wolves of known pedigree. Mol Biol Evol 36(11):2536–2547. 31297530 10.1093/molbev/msz159 PMC6805234

[CR19] Kolesnikov A, Goel S, Nattestad M, Yun T, Baid G, Yang H et al. (2021) DeepTrio: variant calling in families using deep learning. bioRxiv 2021 04(05):438434. 10.1101/2021.04.05.438434.

[CR20] Kucuk E, van der Sanden BPGH, O’Gorman L, Kwint M, Derks R, Wenger AM et al. (2023) Comprehensive de novo mutation discovery with HiFi long-read sequencing. Genome Med 15(1):34. 37158973 10.1186/s13073-023-01183-6 PMC10169305

[CR21] Lex A, Gehlenborg N, Strobelt H, Vuillemot R, Pfister H (2014) UpSet: visualization of Intersecting Sets. IEEE Trans Vis Comput Graph 20(12):1983–92. 26356912 10.1109/TVCG.2014.2346248 PMC4720993

[CR22] Li H (2013) Aligning sequence reads, clone sequences and assembly contigs with BWA-MEM. arXiv 1303.3997. Available from: 10.48550/arXiv.1303.3997

[CR23] Li H (2018) Minimap2: pairwise alignment for nucleotide sequences. Bioinformatics 34(18):3094–3100. 29750242 10.1093/bioinformatics/bty191 PMC6137996

[CR24] Li H, Handsaker B, Wysoker A, Fennell T, Ruan J, Homer N et al. (2009) The sequence alignment/map format and SAMtools. Bioinformatics 25:2078–2079. 19505943 10.1093/bioinformatics/btp352 PMC2723002

[CR25] Liang X, Yang S, Wang D, Knief U (2024) Characterization and distribution of de novo mutations in the zebra finch. Commun Biol 7(1):1243. 39358581 10.1038/s42003-024-06945-5 PMC11447093

[CR26] Logsdon GA, Vollger MR, Eichler EE (2020) Long-read human genome sequencing and its applications. Nat Rev Genet 21(10):597–614. 32504078 10.1038/s41576-020-0236-x PMC7877196

[CR27] López-Cortegano E, Chebib J, Jonas A, Vock A, Künzel S (2025) The rate and spectrum of new mutations in mice inferred by long-read sequencing. Genome Res 35(1):43–54. 39622636 10.1101/gr.279982.124 PMC11789640

[CR28] Lynch M (2010) Evolution of the mutation rate. Trends Genet 26(8):345–352. 20594608 10.1016/j.tig.2010.05.003 PMC2910838

[CR29] Lynch M, Ali F, Lin T, Wang Y, Ni J et al. (2023) The divergence of mutation rates and spectra across the Tree of Life. EMBO Rep 24(10):e57561. 37615267 10.15252/embr.202357561 PMC10561183

[CR30] Mahmoud M, Gobet N, Cruz-Dávalos DI, Mounier N, Dessimoz C, Sedlazeck FJ (2019) Structural variant calling: the long and the short of it. Genome Biol 20(1):246. 31747936 10.1186/s13059-019-1828-7 PMC6868818

[CR31] Malinsky M, Svardal H, Tyers AM, Miska EA, Genner MJ, Turner GF et al. (2018) Whole-genome sequences of Malawi cichlids reveal multiple radiations interconnected by gene flow. Nat Ecol Evol 2(12):1940–1955. 30455444 10.1038/s41559-018-0717-x PMC6443041

[CR32] McKenna A, Hanna M, Banks E, Sivachenko A, Cibulskis K, Kernytsky A et al. (2010) The Genome Analysis Toolkit: a MapReduce framework for analyzing next-generation DNA sequencing data. Genome Res 20(9):1297–1303. 20644199 10.1101/gr.107524.110 PMC2928508

[CR33] Noyes MD, Harvey WT, Porubsky D, Sulovari A, Li R et al. (2022) Familial long-read sequencing increases yield of de novo mutations. Am J Hum Genet 109(4):631–646. 35290762 10.1016/j.ajhg.2022.02.014 PMC9069071

[CR34] Nurk S, Koren S, Rhie A, Rautiainen M, Bzikadze AV, Mikheenko A et al. (2022) The complete sequence of a human genome. Science 376(6588):44–53. 35357919 10.1126/science.abj6987 PMC9186530

[CR35] Pauper M, Kucuk E, Wenger AM, Chakraborty S, Baybayan P, Kwint M et al. (2021) Long-read trio sequencing of individuals with unsolved intellectual disability. Eur J Hum Genet 29(4):637–648. 33257779 10.1038/s41431-020-00770-0 PMC8115091

[CR36] Poplin R, Chang P-C, Alexander D, Schwartz S, Colthurst T, Ku A et al. (2018) A universal SNP and small-indel variant caller using deep neural networks. Nat Biotechnol 36(10):983–987. 30247488 10.1038/nbt.4235

[CR37] Porubsky D, Dashnow H, Sasani TA, Logsdon GA, Hallast P, Noyes MD et al. (2025) Human de novo mutation rates from a four-generation pedigree reference. Nature 643(8071):427–436. 40269156 10.1038/s41586-025-08922-2 PMC12240836

[CR38] Powers D (2011) Evaluation: from precision, recall and F-measure to ROC, informedness, markedness & correlation. J Mach Learn Technol 2(1):37–63.

[CR39] Roach JC, Glusman G, Smit AFA, Huff CD, Hubley R, Shannon PT et al. (2010) Analysis of genetic inheritance in a family quartet by whole genome sequencing. Science 328(5978):636–639. 20220176 10.1126/science.1186802 PMC3037280

[CR40] Robinson JT, Thorvaldsdóttir H, Winckler W, Guttman M, Lander ES, Getz G et al. (2011) Integrative genomics viewer. Nat Biotechnol 29(1):24–26. 21221095 10.1038/nbt.1754 PMC3346182

[CR41] Sendell-Price AT, Tulenko FJ, Pettersson M, Kang D, Montandon M, Winkler S et al. (2023) Low mutation rate in epaulette sharks is consistent with a slow rate of evolution in sharks. Nat Commun 14(1):6628. 37857613 10.1038/s41467-023-42238-x PMC10587355

[CR42] Smith CT, Elfstrom CM, Seeb LW, Seeb JE (2005) Use of sequence data from rainbow trout and Atlantic salmon for SNP detection in Pacific salmon. Mol Ecol 14(13):4193–4203. 16262869 10.1111/j.1365-294X.2005.02731.x

[CR43] Tatsumoto S, Go Y, Fukuta K, Noguchi H, Hayakawa T, Tomonaga M et al. (2017) Direct estimation of de novo mutation rates in a chimpanzee parent-offspring trio by ultra-deep whole genome sequencing. Sci Rep 7(1):13561. 29093469 10.1038/s41598-017-13919-7 PMC5666008

[CR44] Versoza CJ, Ehmke EE, Jensen JD, Pfeifer SP (2025) Characterizing the rates and patterns of de novo germline mutations in the aye-aye (Daubentonia madagascariensis). Mol Biol Evol 42(3):msaf034. 40048663 10.1093/molbev/msaf034 PMC11884812

[CR45] Wang X, Wang D, Wang H, Dudgeon D, Reid K, Merilä J (2025) Chromosome-level haplotype-resolved genome of the tropical loach (*Oreonectes platycephalus*). Sci Data 12(1):29. 39774106 10.1038/s41597-024-04301-0 PMC11707185

[CR46] Wang Y, Obbard DJ (2023) Experimental estimates of germline mutation rate in eukaryotes: a phylogenetic meta-analysis. Evol Lett 7(4):216–226. 37475753 10.1093/evlett/qrad027 PMC10355183

[CR47] Wooldridge TB, Ford SM, Conwell HC, Hyde J, Harris K, Shapiro B (2025) Direct measurement of the mutation rate and its evolutionary consequences in a critically endangered mollusk. Mol Biol Evol 42(1):msae266. 39775835 10.1093/molbev/msae266 PMC11704959

[CR48] Ye J, Coulouris G, Zaretskaya I, Cutcutache I, Rozen S, Madden TL (2012) Primer-BLAST: a tool to design target-specific primers for polymerase chain reaction. BMC Bioinform 13: 134. 10.1186/1471-2105-13-134 PMC3412702 22708584

[CR49] Yoder AD, Tiley GP (2021) The challenge and promise of estimating the de novo mutation rate from whole-genome comparisons among closely related individuals. Mol Ecol 30(23):6087–6100. 34062029 10.1111/mec.16007

[CR50] Yun T, Li H, Chang PC, Lin MF, Carroll A, McLean CY (2021) Accurate, scalable cohort variant calls using DeepVariant and GLnexus. Bioinformatics 36(24):5582–5589. 33399819 10.1093/bioinformatics/btaa1081 PMC8023681

[CR51] Zhang C, Reid K, Sands AF, Fraimout A, Schierup MH, Merilä J (2023) De novo mutation rates in sticklebacks. Mol Biol Evol 40(9):msad192. 37648662 10.1093/molbev/msad192 PMC10503787

[CR52] Zhang C, Reid K, Schierup MH, Wang H, Candolin U, Merilä J (2025) Rate of de novo mutations in the three-spined stickleback. Heredity 134(7):387–395. 40506496 10.1038/s41437-025-00767-9 PMC12218844

[CR53] Zhou Y, Zhang Z, Bao Z, Li H, Lyu Y, Zan Y et al. (2022) Graph pangenome captures missing heritability and empowers tomato breeding. Nature 606(7914):527–534. 35676474 10.1038/s41586-022-04808-9 PMC9200638

