# Supplementary material for: How precise are mutation rate estimates? Comparison of different approaches to estimate de novo mutation rates
Source: Heredity (Edinb). 2026 Jun 19;135(6):445–52. doi: 10.1038/s41437-026-00852-7 (PMC13354566; doi:10.1038/s41437-026-00852-7)
Supplement: Supplementary file 2 — Supporting information [file 41437_2026_852_MOESM2_ESM.docx]

### Supporting information for:

**How precise are mutation rate estimates? Comparison of different approaches to estimate de novo mutation rates**

Table of Contents:

| Figure S1 | Page 2 |
| --- | --- |
| Figure S2 | Page 3 |
| Figure S3 | Page 4 |
| Figure S4 | Page 5 |
| Supplemental Material | Page 6 |


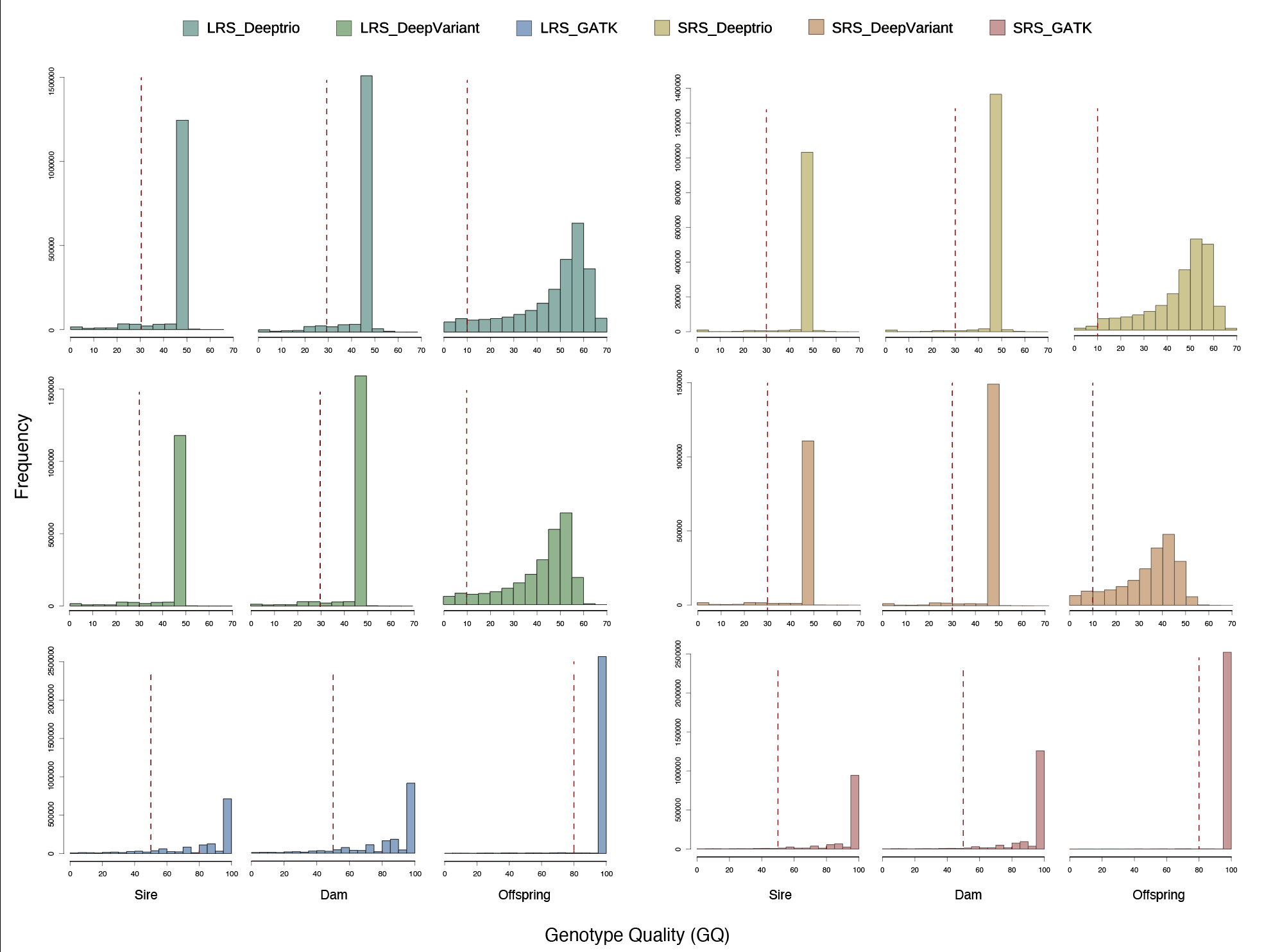
Figure S1. Distribution of Genotype Quality (GQ) of different approaches. The red line represents the filtering threshold (details are provided in the methods).


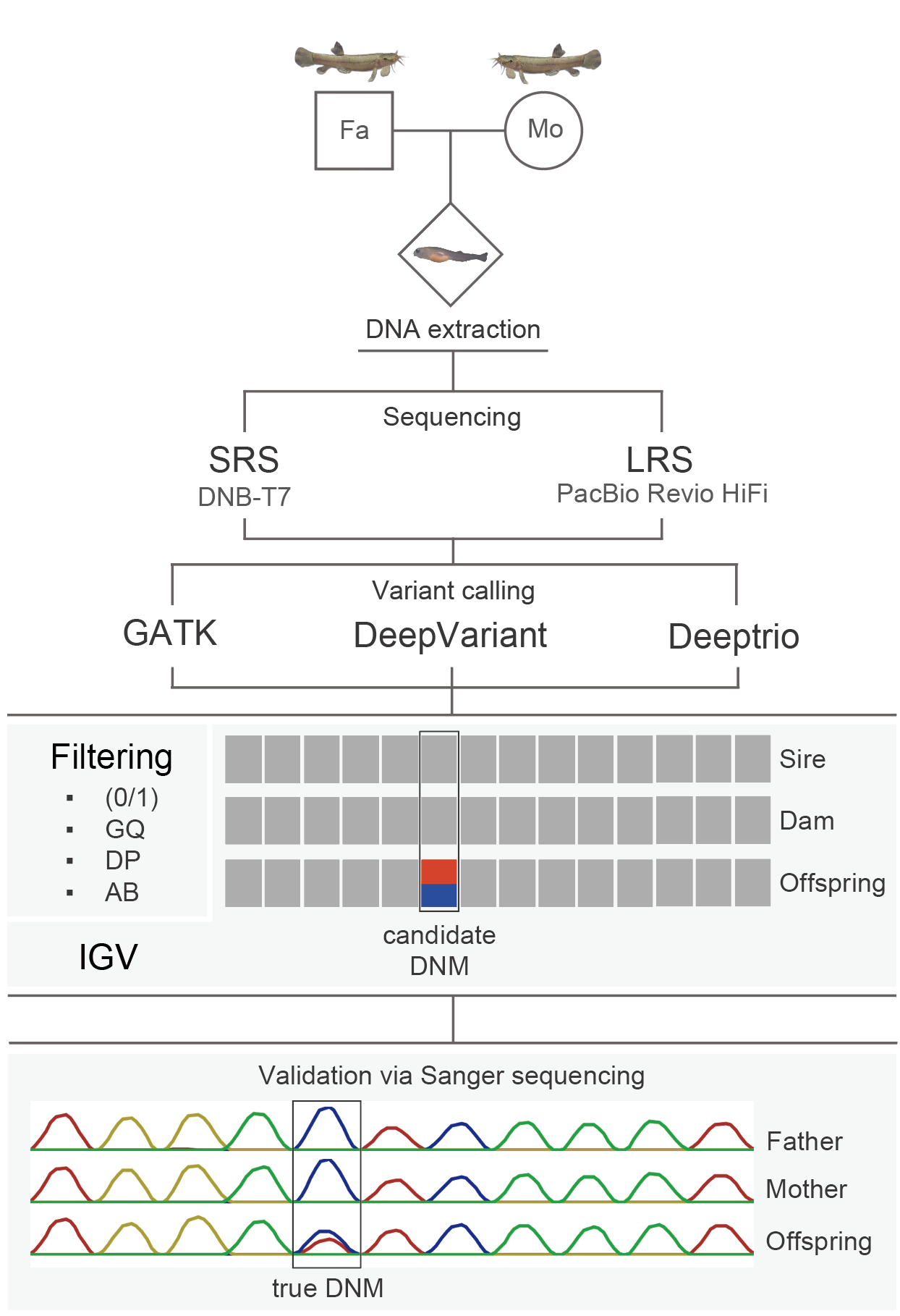


Figure S2. *De novo* DNM calling and validation workflow.


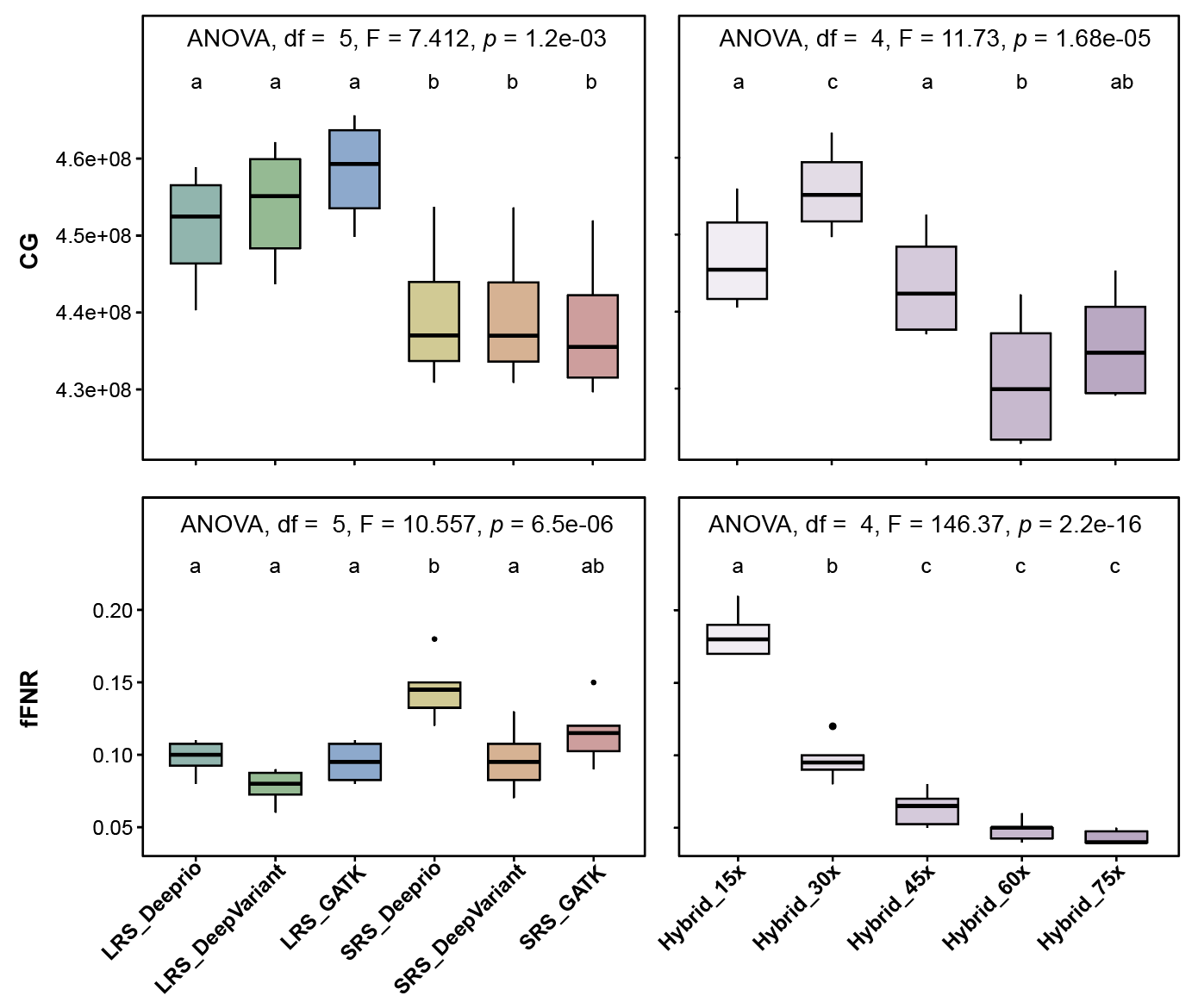


Figure S3. Callable genome (CG) and fFNR obtained using different approaches at 30x (left) or under different mapping depths (right). Boxes represent interquartile ranges (IQR), horizontal lines denote medians, whiskers extend to 1.5×IQR, and dots indicate outliers. Letters above each box indicate results of Tukey HSD post-hoc tests (α = 0.05), where identical letters denote no significant difference between groups (p > 0.05), and distinct letters indicate significant differences (p < 0.05).


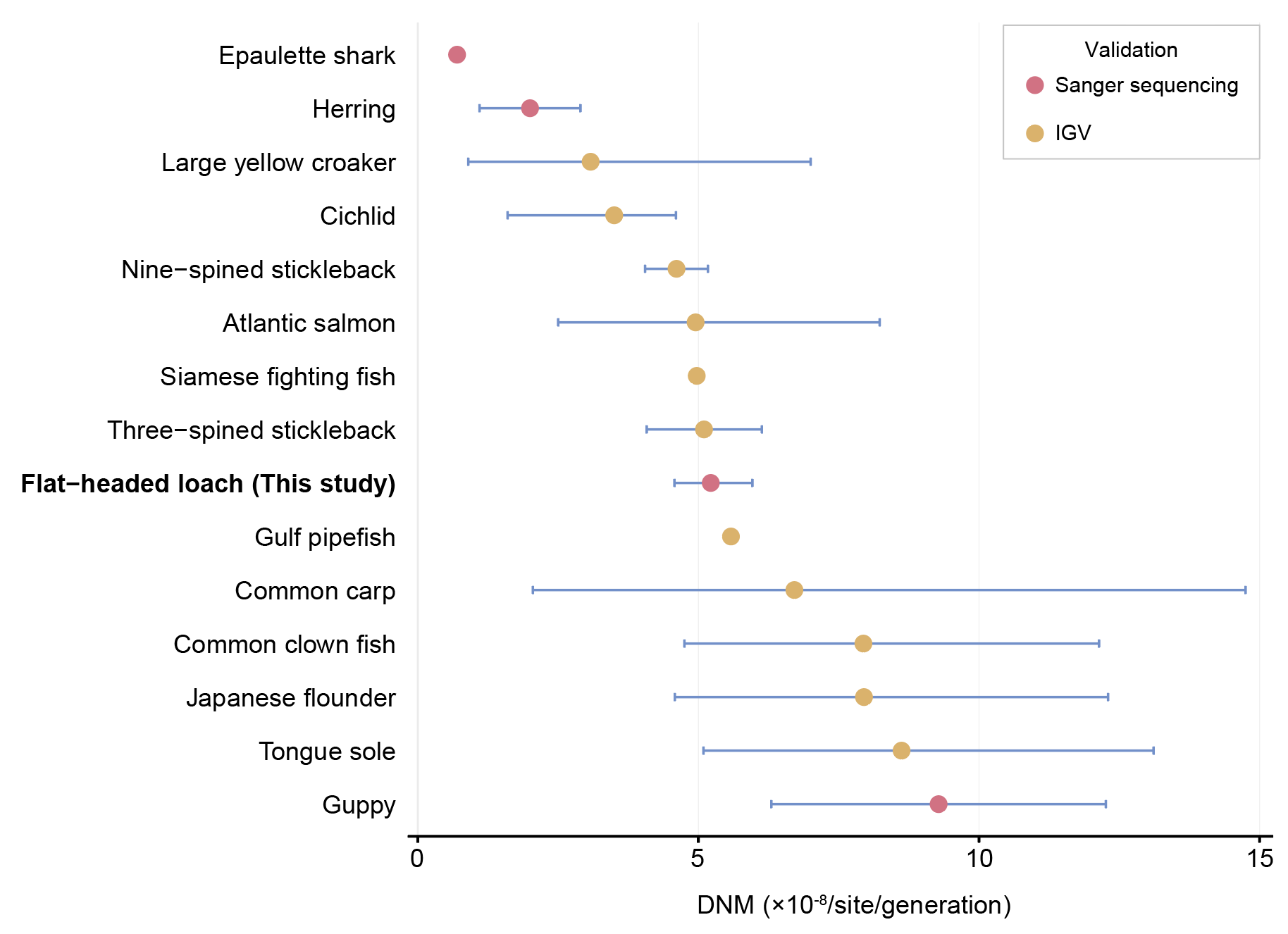


Figure S4. The pedigree-based DNM rate per generation for different fish species. The 95% CIs are shown except for those that are not available. For more details and references, see Table S7.

#### **Supplemental Material**

**IGV checking**

A true *de novo* mutation must meet trio segregation (present in offspring and absent in parents) and the allele frequency of the mutation of the offspring must be between 0.3 and 0.7. Two situations were found in IGV (Figure 1): a) Parents were homozygous and the offspring was heterozygous; b) parents were homozygous and the offspring was heterozygous, but so many mutations within 100bp and all the mutations were located on the same read. Given that mutation is a rare event, situation 2 could reflect mismapping of similar reads rather than a DNM event. Therefore, only situation 1 was considered as a DNM candidate.


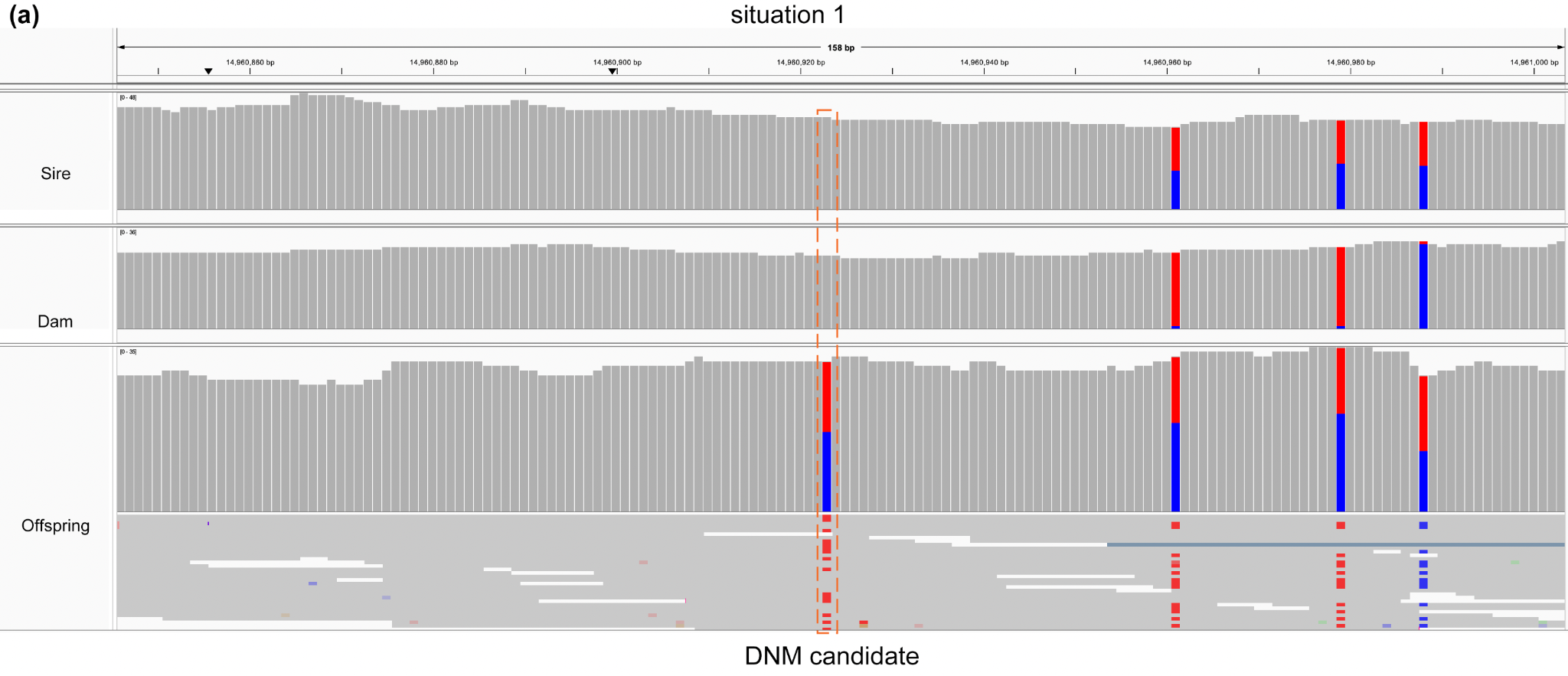


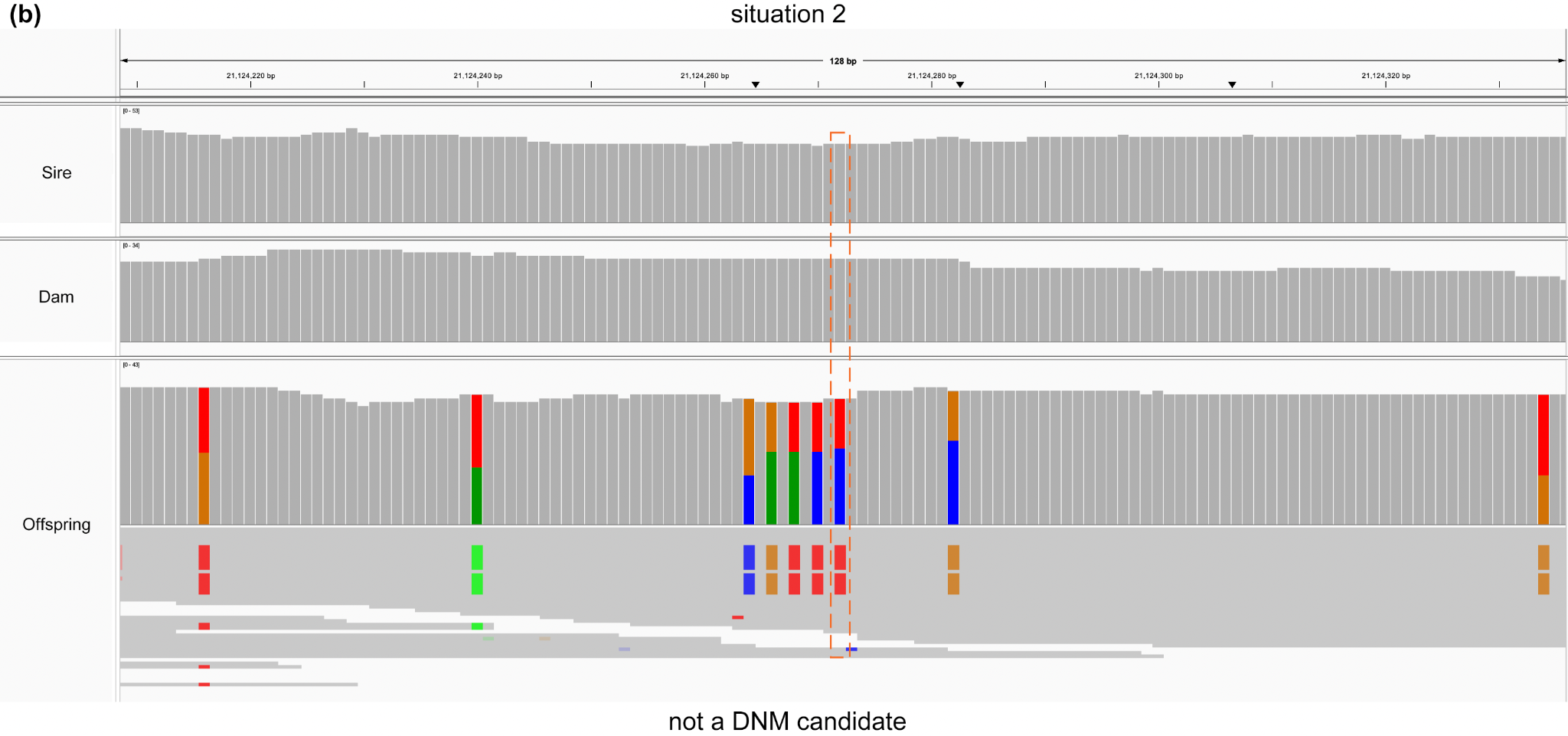


Figure 1. Identify DNM candidates via IGV.

**Sanger sequencing**

Before Sanger sequencing, PCR amplification was performed on the sampled trio. The total PCR volume was 30 μl, comprising 50 ng DNA template, 0.4 μM primers, 15 μl of 2× Taq PCR StarMix with loading dye, and 8 μl of deionized water. The PCR program was as follows: initial denaturation at 95°C for 5 min, followed by 30 cycles of denaturation at 95°C for 30 s, annealing at 57°C for 30 s, and extension at 72°C for 45 s, with a final extension at 72°C for 5 min.

After receiving the pair-end sequencing data, the traces were analysed using Geneious v2025.0.3 (Kearse et al. 2012). There were three situations (Figure 2): 1) Parents were homozygous and the offspring was heterozygous - true DNM; 2) parents and offspring were homozygous which indicated a false positive signal; 3) one of the parents was heterozygous (richness too low to be detected by IGV) so the heterozygosity of the offspring was inherited - also a false positive signal.


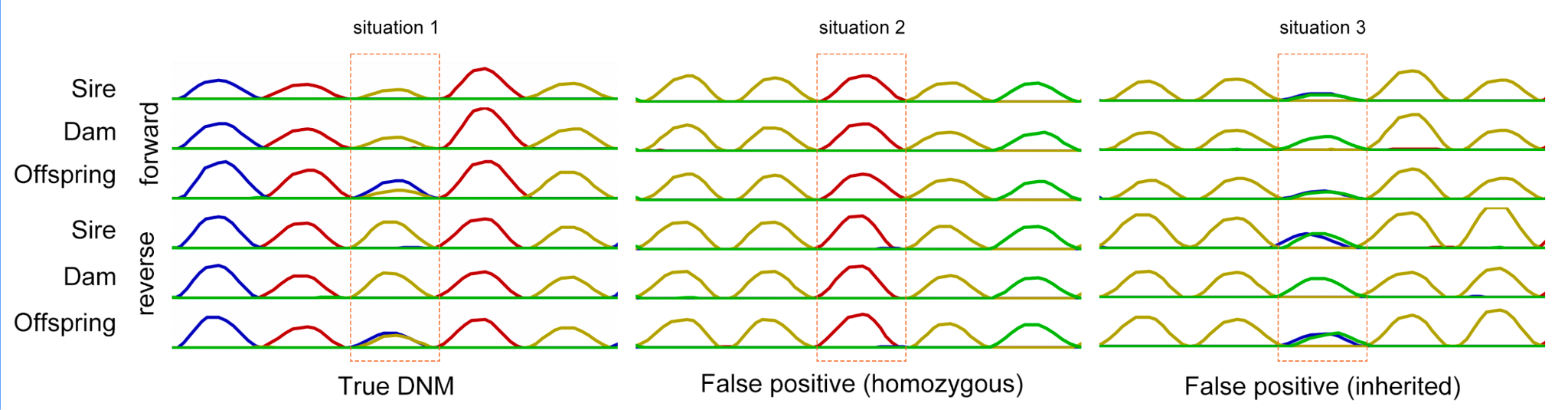


Figure 2. Validate DNMs via Sanger sequencing.

**ANOVA for callable genome (CG) and filtering false negative rate (fFNR)**

To dissect the factors underlying this discrepancy, one-way ANOVA was performed on CG and fFNR obtained under different mapping depths or using different approaches at 30x. ANOVA was conducted with mapping depth / different approaches (Table S6) as fixed factors and CG/fFNR as the dependent variable, with statistical significance set at *p* < 0.05. Post-hoc multiple comparisons were further carried out using Tukey’s honest significant difference (HSD) test to identify pairwise differences in CG and fFNR values across the tested groups, and Benjamini-Hochberg correction was applied to adjust for multiple testing errors.

**Literature search**

To better understand methods for minimizing false positives (FPs), we conducted a literature search of de novo mutation studies using the parent-offspring (PO) method. According to Wang & Obbard (2023), 32 papers estimated the *de* novo mutation rate in non-human species using the PO method. In addition, using Google Scholar, we searched for publications containing the terms "*de novo*/germline, mutation rate" and limited the search to years after 2023 (including preprint studies). In total, 38 studies were retained for discussion (Table S1, References), of which eight were related to fish species (Table S1 & S7).

**DNM detection in the other family**

DNM calling for the other family (including 13 trios, 30x) was performed using the SRS_DeepVariant method, following the standard pipeline described in the main text, with the same filtration criteria. The remaining candidate DNMs were manually inspected in IGV, with only high-confidence DNMs retained for Sanger sequencing. After Sanger sequencing, 44 (out of 55) DNMs were detected, with 21 transitions and 23 transversions (Ts/Tv = 0.913).

**References**

Armstrong EE, Carey SB, Harkess A, Zenato Lazzari G, Solari KA, Maldonado JE, *et al.* (2025). Parameterizing Pantherinae: De Novo Mutation Rate Estimates from Panthera and Neofelis Pedigrees. *Genome Biology and Evolution* **17**: evaf060.

Baranova MA, Logacheva MD, Penin AA, Seplyarskiy VB, Safonova YY, Naumenko SA, *et al.* (2015). Extraordinary Genetic Diversity in a Wood Decay Mushroom. *Molecular Biology and Evolution* **32**: 2775–2783.

Bergeron LA, Besenbacher S, Bakker J, Zheng J, Li P, Pacheco G, *et al.* (2021). The germline mutational process in rhesus macaque and its implications for phylogenetic dating. *GigaScience* **10**: giab029.

Bergeron LA, Besenbacher S, Zheng J, Li P, Bertelsen MF, Quintard B, *et al.* (2023). Evolution of the germline mutation rate across vertebrates. *Nature* **615**: 285–291.

Besenbacher S, Hvilsom C, Marques-Bonet T, Mailund T, Schierup MH (2019). Direct estimation of mutations in great apes reconciles phylogenetic dating. *Nat Ecol Evol* **3**: 286–292.

Burda K, Konczal M (2023). Validation of machine learning approach for direct mutation rate estimation. *Molecular Ecology Resources* **23**: 1757–1771.

Campbell CR, Tiley GP, Poelstra JW, Hunnicutt KE, Larsen PA, Lee H-J, *et al.* (2021). Pedigree-based and phylogenetic methods support surprising patterns of mutation rate and spectrum in the gray mouse lemur. *Heredity* **127**: 233–244.

Feng C, Pettersson M, Lamichhaney S, Rubin C-J, Rafati N, Casini M, *et al.* (2017). Moderate nucleotide diversity in the Atlantic herring is associated with a low mutation rate (M Przeworski, Ed.). *eLife* **6**: e23907.

Han M, Ren J, Guo H, Tong X, Hu H, Lu K, *et al.* (2023). Mutation Rate and Spectrum of the Silkworm in Normal and Temperature Stress Conditions. *Genes (Basel)* **14**: 649.

Keightley PD, Ness RW, Halligan DL, Haddrill PR (2014). Estimation of the Spontaneous Mutation Rate per Nucleotide Site in a Drosophila melanogaster Full-Sib Family. *Genetics* **196**: 313–320.

Keightley PD, Pinharanda A, Ness RW, Simpson F, Dasmahapatra KK, Mallet J, *et al.* (2015). Estimation of the Spontaneous Mutation Rate in Heliconius melpomene. *Molecular Biology and Evolution* **32**: 239–243.

Kearse M, Moir R, Wilson A, Stones-Havas S, Cheung M, Sturrock S, Buxton S, Cooper A, Markowitz S, Duran C, et al. 2012. Geneious Basic: an integrated and extendable desktop software platform for the organization and analysis of sequence data. *Bioinforma*. Oxf. Engl. **28**:1647–1649.

Koch EM, Schweizer RM, Schweizer TM, Stahler DR, Smith DW, Wayne RK, *et al.* (2019). De Novo Mutation Rate Estimation in Wolves of Known Pedigree. *Molecular Biology and Evolution* **36**: 2536–2547.

Krasovec M (2021). The spontaneous mutation rate of Drosophila pseudoobscura. *G3 Genes|Genomes|Genetics* **11**: jkab151.

Krasovec M, Chester M, Ridout K, Filatov DA (2018). The Mutation Rate and the Age of the Sex Chromosomes in *Silene latifolia*. *Current Biology* **28**: 1832-1838.e4.

Liang X, Yang S, Wang D, Knief U (2024). Characterization and distribution of de novo mutations in the zebra finch. *Commun Biol* **7**: 1–13.

Lin Y, Darolti I, Bijl W van der, Morris J, Mank JE (2023). Extensive variation in germline de novo mutations in Poecilia reticulata. *Genome Res* **33**: 1317–1324.

Liu H, Jia Y, Sun X, Tian D, Hurst LD, Yang S (2017). Direct Determination of the Mutation Rate in the Bumblebee Reveals Evidence for Weak Recombination-Associated Mutation and an Approximate Rate Constancy in Insects. *Molecular Biology and Evolution* **34**: 119–130.

Malinsky M, Svardal H, Tyers AM, Miska EA, Genner MJ, Turner GF, *et al.* (2018). Whole-genome sequences of Malawi cichlids reveal multiple radiations interconnected by gene flow. *Nat Ecol Evol* **2**: 1940–1955.

Martin HC, Batty EM, Hussin J, Westall P, Daish T, Kolomyjec S, *et al.* (2018). Insights into Platypus Population Structure and History from Whole-Genome Sequencing. *Molecular Biology and Evolution* **35**: 1238–1252.

Peña-Garcia Y, Wang RJ, Raveendran M, Harris RA, Samollow PB, Rogers J, *et al.* (2024). Low mutation rate but high male-bias in the germline of a short-lived opossum. : 2024.12.05.627076.

Pfeifer SP (2017). Direct estimate of the spontaneous germ line mutation rate in African green monkeys. *Evolution* **71**: 2858–2870.

Popovic I, Bergeron LA, Bozec Y-M, Waldvogel A-M, Howitt SM, Damjanovic K, *et al.* (2024). High germline mutation rates, but not extreme population outbreaks, influence genetic diversity in a keystone coral predator. *PLOS Genetics* **20**: e1011129.

Póti Á, Szüts D, Vermezovic J (2024). Mutational profile of the regenerative process and de novo genome assembly of the planarian Schmidtea polychroa. *Nucleic Acids Research* **52**: 1779–1792.

Rashid I, Campos M, Collier T, Crepeau M, Weakley A, Gripkey H, *et al.* (2022). Spontaneous mutation rate estimates for the principal malaria vectors Anopheles coluzzii and Anopheles stephensi. *Sci Rep* **12**: 226.

Recknagel H, Elmer KR, Meyer A (2013). A Hybrid Genetic Linkage Map of Two Ecologically and Morphologically Divergent Midas Cichlid Fishes (Amphilophus spp.) Obtained by Massively Parallel DNA Sequencing (ddRADSeq). *G3 (Bethesda)* **3**: 65–74.

Rochus CM, Steensma MJ, Bink MCAM, Huisman AE, Harlizius B, Derks MFL, Crooijmans RPMA, Ducro BJ, Bijma P, Groenen MAM, Mulder HA (2025). Estimating mutation rate and characterising single nucleotide de novo mutations in pigs. Genet Sel Evol. **57**(1):21.

Sendell-Price AT, Tulenko FJ, Pettersson M, Kang D, Montandon M, Winkler S, *et al.* (2023). Low mutation rate in epaulette sharks is consistent with a slow rate of evolution in sharks. *Nat Commun* **14**: 6628.

Smeds L, Qvarnström A, Ellegren H (2016). Direct estimate of the rate of germline mutation in a bird. *Genome Res* **26**: 1211–1218.

Tatsumoto S, Go Y, Fukuta K, Noguchi H, Hayakawa T, Tomonaga M, *et al.* (2017). Direct estimation of de novo mutation rates in a chimpanzee parent-offspring trio by ultra-deep whole genome sequencing. *Sci Rep* **7**: 13561.

Thomas GWC, Wang RJ, Puri A, Harris RA, Raveendran M, Hughes DST, *et al.* (2018). Reproductive Longevity Predicts Mutation Rates in Primates. *Current Biology* **28**: 3193-3197.e5.

Versoza CJ, Ehmke EE, Jensen JD, Pfeifer SP (2025). Characterizing the Rates and Patterns of De Novo Germline Mutations in the Aye-Aye (Daubentonia madagascariensis). *Molecular Biology and Evolution* **42**: msaf034.

Wang RJ, Peña-Garcia Y, Bibby MG, Raveendran M, Harris RA, Jansen HT, *et al.* (2022). Examining the Effects of Hibernation on Germline Mutation Rates in Grizzly Bears. *Genome Biology and Evolution* **14**: evac148.

Wang RJ, Raveendran M, Harris RA, Murphy WJ, Lyons LA, Rogers J, *et al.* (2022). De novo Mutations in Domestic Cat are Consistent with an Effect of Reproductive Longevity on Both the Rate and Spectrum of Mutations. *Molecular Biology and Evolution* **39**: msac147.

Wang RJ, Thomas GWC, Raveendran M, Harris RA, Doddapaneni H, Muzny DM, *et al.* (2020). Paternal age in rhesus macaques is positively associated with germline mutation accumulation but not with measures of offspring sociability. *Genome Res* **30**: 826–834.

Wang Y, McNeil P, Abdulazeez R, Pascual M, Johnston SE, Keightley PD, *et al.* (2023). Variation in mutation, recombination, and transposition rates in Drosophila melanogaster and Drosophila simulans. *Genome Res* **33**: 587–598.

Wang Y, Obbard DJ (2023). Experimental estimates of germline mutation rate in eukaryotes: a phylogenetic meta-analysis. *Evolution Letters* **7**: 216–226.

Wooldridge TB, Ford SM, Conwell HC, Hyde J, Harris K, Shapiro B (2025). Direct Measurement of the Mutation Rate and Its Evolutionary Consequences in a Critically Endangered Mollusk. *Molecular Biology and Evolution* **42**: msae266.

Yang S, Wang L, Huang J, Zhang X, Yuan Y, Chen J-Q, *et al.* (2015). Parent–progeny sequencing indicates higher mutation rates in heterozygotes. *Nature* **523**: 463–467.

Yang N, Xu XW, Wang RR, Peng WL, Cai L, Song J-M, *et al.* (2017). Contributions of Zea mays subspecies mexicana haplotypes to modern maize. *Nat Commun* **8**: 1874.

Yang C, Zhou Y, Marcus S, Formenti G, Bergeron LA, Song Z, *et al.* (2021). Evolutionary and biomedical insights from a marmoset diploid genome assembly. *Nature* **594**: 227–233.

Zhang SJ, Ma J, Riera M, Besenbacher S, Niskanen J, Salokorpi N, *et al.* (2025). Determinants of de novo mutations in extended pedigrees of 43 dog breeds. *Genome Biol.* **26**(1):305.

Zhang C, Reid K, Sands AF, Fraimout A, Schierup MH, Merilä J (2023). De Novo Mutation Rates in Sticklebacks. *Molecular Biology and Evolution* **40**: msad192.

Zhang C, Reid K, Schierup MH, Wang H, Candolin U, Merilä J (2025). Rate of de novo mutations in the three-spined stickleback. *Heredity* **134**: 387–395.

Zhang M, Yang Q, Ai H, Huang L (2022). Revisiting the Evolutionary History of Pigs via *De Novo* Mutation Rate Estimation in A Three-generation Pedigree. *Genomics, Proteomics & Bioinformatics* **20**: 1040–1052.
